# Supplementary material for: Fatal iatrogenic cerebral β-amyloid-related arteritis in a woman treated with lecanemab for Alzheimer’s disease
Source: Nat Commun. 2023 Dec 12;14:8220. doi: 10.1038/s41467-023-43933-5 (PMC10716177; doi:10.1038/s41467-023-43933-5)
Supplement: Supplementary file 1 — Supplementary Information [file 41467_2023_43933_MOESM1_ESM.pdf]

Supplementary information

Table of Contents

1. Expanded case history

Initial presentation

Medical history

Examination

Diagnostic evaluations

Hospital course

Autopsy

2. Neuroimaging supplement

Supplementary Figure 1. Montage of FLAIR images from pre-event MRI.

Supplementary Figure 2. Montage of FLAIR images from post-event MRI.

Supplementary Figure 3. Montage of GRE-T2* images from pre-event MRI.

Supplementary Figure 4. Montage of GRE-T2* images from post-event MRI.

Supplementary Figure 5. Selected images from diffusion weighted scan (post-event).

Supplementary Figure 6. Selected images from β-amyloid PET and tau PET scans.

3. Neuropathological supplement

Supplementary Figure 7. Locations of tissue blocks for neuropathology examination.

Supplementary Figure 8. β-amyloid and tau pathology.

1. Expanded case history

Initial presentation: The patient was 79 years old and in good general health prior to the admission in question. She had early-stage Alzheimer's disease, no clinical history of hypertension and took fluoxetine for mild depression, omeprazole for gastroesophageal reflux, along with atorvastatin and vitamins. The patient's study partner reported that she complained of headaches and "brain fog" for the last several weeks. The headaches were most severe immediately after infusions of an experimental drug in a clinical trial and lasted one to two days during which she mostly stayed in bed. Her study partner additionally reported that in the days prior to the precipitating event she was more easily disoriented than typical. On the day in question, she was brought by EMS to the hospital for what her study partner described as a seizure. While eating at a restaurant, she looked and turned to the left, became stiff and her speech became slurred. She then had 30 seconds of generalized convulsion after which she slumped out of her chair due to flaccid left hemiplegia. When the EMS team evaluated her, her Glasgow Coma Scale was less than 8 and she was hypoxic (SpO2 ~80%) prompting intubation in the field with fentanyl and midazolam. Upon arrival in the ER her blood pressure was 126/99, rapid glucose screen was normal. She was sedated with propofol infusion. An emergent CT scan of the head was interpreted as: "extensive low-density within the periventricular region bilaterally; no intracranial hemorrhage, mass or acute territorial infarcts." The treating team initially felt her presentation was most worrisome for a stroke. The ER team and consulting neurologist considered administering a thrombolytic agent but decided to forego this treatment based on the unclear history and suboptimal neurological exam due to sedation from the recent intubation. The family informed

the treating team that the patient “was getting some experimental medication for dementia at a research center -- she got 3 doses in the last six weeks.”

Medical history: She had mild dementia, but was independent, living with a roommate who was also her study partner. She participated in a randomized trial of an anti-β-amyloid immunotherapy called lecanemab for approximately 18 months during which time she was receiving the placebo. She felt no obvious side effects nor obvious benefits during the randomized portion of the study. She then enrolled in the open-label extension of the trial where she was guaranteed to receive the active drug and had received three infusions (10mg/kg) at two-week intervals. Preconditions of the study included documentation of pathological levels of β-amyloid by PET imaging; her imaging prior to enrollment in the open label extension continued to show amyloid positivity (see supplementary figure 6). Her other medical problems were gastroesophageal reflux, right knee replacement and a small meningioma which had been stable in size for decades. She notably had no prior history of seizures, no tobacco, alcohol, or sedative use.

Examination: Her initial examination upon arrival to the hospital was limited due to recent intubation, but once admitted to the ICU and sedatives were paused, she was unable to follow commands and was agitated, but regained motor function on the left as she was noted to withdraw from noxious stimulation symmetrically in all extremities. She had no obvious cranial nerve defect.

Diagnostic evaluations: Initial labs were notable for leukocytosis (22,100/mm^3 with a 92% neutrophilic predominance) which returned to nearly normal within 24 hours. Platelet count was normal (205,000/mm^3), and she was not anemic (Hgb 12.8 g/dL). A chemistry panel showed normal renal function and normal liver function tests, but she developed elevated transaminases on hospital day 2 (AST 109 IU/L, ALT 78 IU/L, alkaline phosphatase 101 U/L, and total bilirubin 0.8 mg/dL). All sputum, blood and urine cultures were negative during the hospitalization.

Her EKG showed she was in atrial fibrillation. Echocardiogram was normal (EF 55%, normal valves, wall motion and chamber dimensions). Her apolipoprotein genotype was homozygous APOE4/4.

EEG on hospital day 1 interpreted as follows: No posterior dominant rhythm; background consisted of 4-5 hz activity that appeared symmetric. There were frontal sharp waves that appeared rhythmic consistent with a FIRDA pattern. Photic stimulation evoked a driving response at certain flash frequencies.

The key sequences from the MRI are available in the attached imaging summary. Post-contrast images were also obtained and were uninformative.

A baseline MRI was obtained before enrollment in the open label extension of the clinical trial (a little more than six weeks prior to her acute event) and is included in this material for additional context.

No CSF analysis was performed.

Hospital course: She was started on heparin infusion after atrial fibrillation was identified on day 1. Anticoagulation was monitored with factor Xa levels – the highest was 2.5x the upper limit of the reference range and this was stopped on day 2. She was also started on ceftriaxone out of concern for aspiration pneumonia. Her home medications of aspirin, atorvastatin, metoprolol and fluoxetine were continued while an inpatient. The patient’s clinical trial physician raised a concern for the possibility of amyloid-related imaging abnormality (ARIA) as a side effect from the experimental drug. She was started on Keppra 500 mg BID and 1 g daily of solumedrol which was continued for four doses over three days. On the third day, the patient was extubated with instructions not to reintubate, in line with requests in her living will. She remained “agitated and restless” according to nursing staff, mute aside from occasional unintelligible mutterings, she was unable to follow commands, but spontaneously moved all extremities. To manage agitation, she was sedated using dexmedetomidine infusions on days 3-5. She was also given 25mg quetiapine on days 3-5 for agitation and occasional doses of lorazepam for anxiolysis to assist with diagnostic testing. She was started on a nicardipine infusion on day three for blood pressure management. Repeat CT head showed neither worsening nor improvement in the white matter hypodensities and no new pathology. At the end of hospital day 3, her breathing deteriorated, and she became more hypoxic. A loop diuretic was administered, and she was placed on high-flow oxygen with bilevel positive airway

pressure. Her antibiotic regimen was broadened to piperacillin/tazobactam. She also required a diltiazem infusion to control tachycardia. She developed an elevated highly sensitive troponin level (504 pg/ml) which was attributed to the rapid atrial fibrillation and downtrended on subsequent testing. Late in hospital day 4, she had an aspiration event, developed respiratory distress and hypotension, and subsequently developed renal injury (Cr 2.1 mg/dL, BUN 48 mg/dL), leukocytosis (20,000/mm^3), and worsened transaminitis (AST 2398 IU/L, ALT 1854 IU/L, alkaline phosphatase 187 U/L, tBili 2.4 mg/dL). Consistent with her wishes to forego invasive life support measures, the family and treating team instituted comfort-oriented care measures and the patient died in about one day.

Autopsy: The general autopsy found bronchopneumonia with diffuse alveolar damage. It also noted atherosclerosis, left ventricular myocardial hypertrophy and nephrosclerosis. Atherosclerotic plaque occupied about 60-70% of the intimal surface of the abdominal aorta and approximately 60% of the proximal anterior descending coronary artery, interpreted as moderate stenosis. The heart weighed 370 grams. Microscopy examination of the heart showed mild to moderate arteriolosclerosis consistent with hypertensive small vessel disease. There was no infarction. Hyalinizing arteriolosclerosis and focal glomerulosclerosis was present in the kidneys.

The brain was removed and immersion fixed in 10% formaldehyde prior to coronal sectioning into roughly 1 cm coronal slabs. For histological analysis, 16 blocks of tissue were paraffin embedded. The locations of the tissue blocks is shown in supplementary figure 7 and include the right middle frontal gyrus, right anterior superior and middle temporal gyrus, the right mid-to-posterior middle and inferior temporal gyri, the left striate region, the right anterior cingulate gyrus with corpus callosum, the right striatum, right basal ganglia at anterior commissure, right thalamus, left mid hippocampus, left amygdala, caudal midbrain/rostral pons, pons, medulla, left cerebellar cortex with dentate nucleus, right parietal cortex (with hemorrhage), and right inferior temporo-parietal cortex with hemorrhages. Microscopic neuropathological analysis was conducted independently by Dr. Coyne and Dr. Harmsen. The results were discussed by consensus conference.

On gross evaluation, the brain weighed 1320 grams. The left cerebral hemisphere was mildly full in comparison to the right, and the cortical gyri were widened and flattened against the leptomeninges, consistent with moderate cerebral cortical edema. The large blood vessels arose normally and were of normal configuration with intermittent nonocclusive atheromatous plaques. Numerous black petechial hemorrhages were appreciated on the surface of the cortex, most prominently in the left temporal lobe but also in the right temporal lobe and in the bilateral parietal and occipital lobes. Additionally, numerous punctate hemorrhages were also found on the cut surfaces of the cortex of all lobes, with the highest concentration of these being in the inferior temporal lobes. No laminar necrosis nor infarctions were found. The bilateral hippocampi were mildly atrophic.

Three blocks of tissue were submitted to Vanderbilt’s VANTAGE genomics core for DNA extraction and sequencing to assess APOE genotype. A control panel was simultaneously genotyped to ensure correct allelic discrimination. All three replicates returned identical results, homozygous C/C at rs429358 and homozygous C/C at rs7412, consistent with a homozygous E4 genotype.

Supplementary Figure 1. Montage of FLAIR images from pre-event MRI.

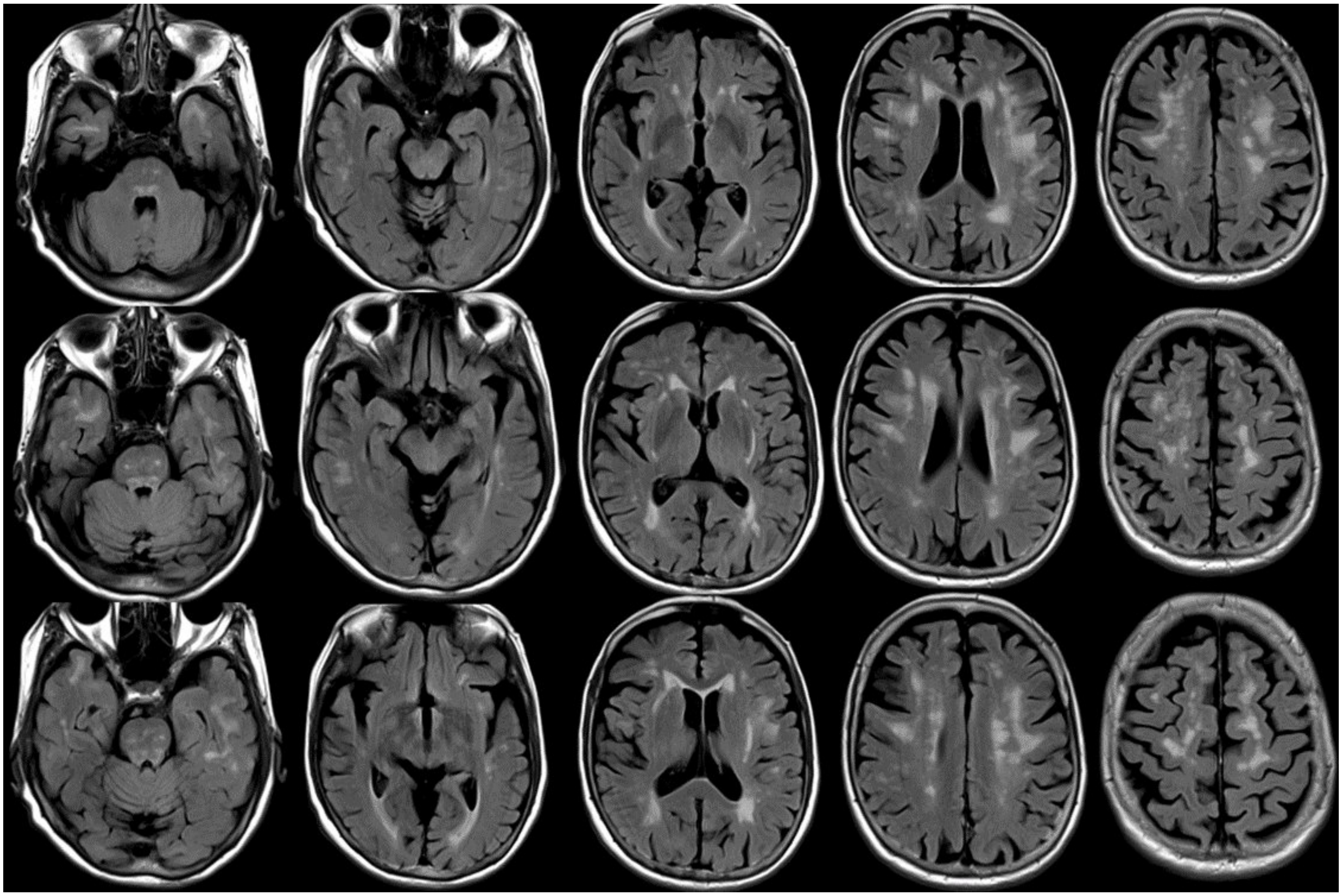

Pre-lecanemab treatment FLAIR imaging (3T field strength, TR 10000 ms, TE 97.0 ms, slice thickness 5.0 mm, interslice gap 0.5 mm) showed moderate, diffuse white matter disease symmetrically involving both hemispheres.

Supplementary Figure 2. Montage of FLAIR images from post-event MRI.

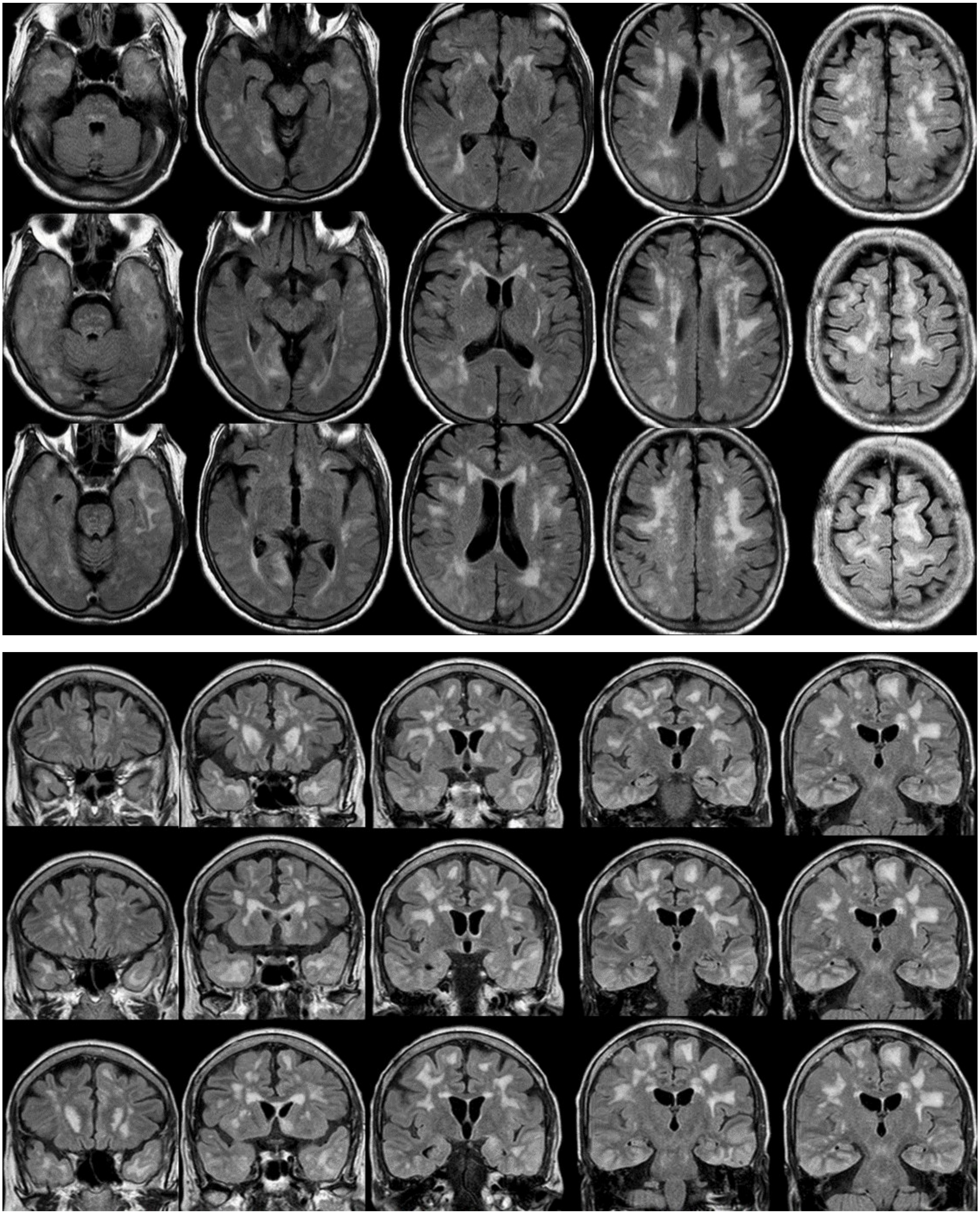

Post-lecanemab treatment FLAIR imaging (1.5T field strength, TR 6000, TE 120.0, slice thickness 5.0mm, interslice gap 1.0mm) showed worsening white matter disease with edema involving primarily the temporal, parietal, and occipital lobes bilaterally. The cerebral edema effaces the sulci on the posterior portions of the brain.

Supplementary Figure 3. Montage of GRE-T2* images from pre-event MRI.

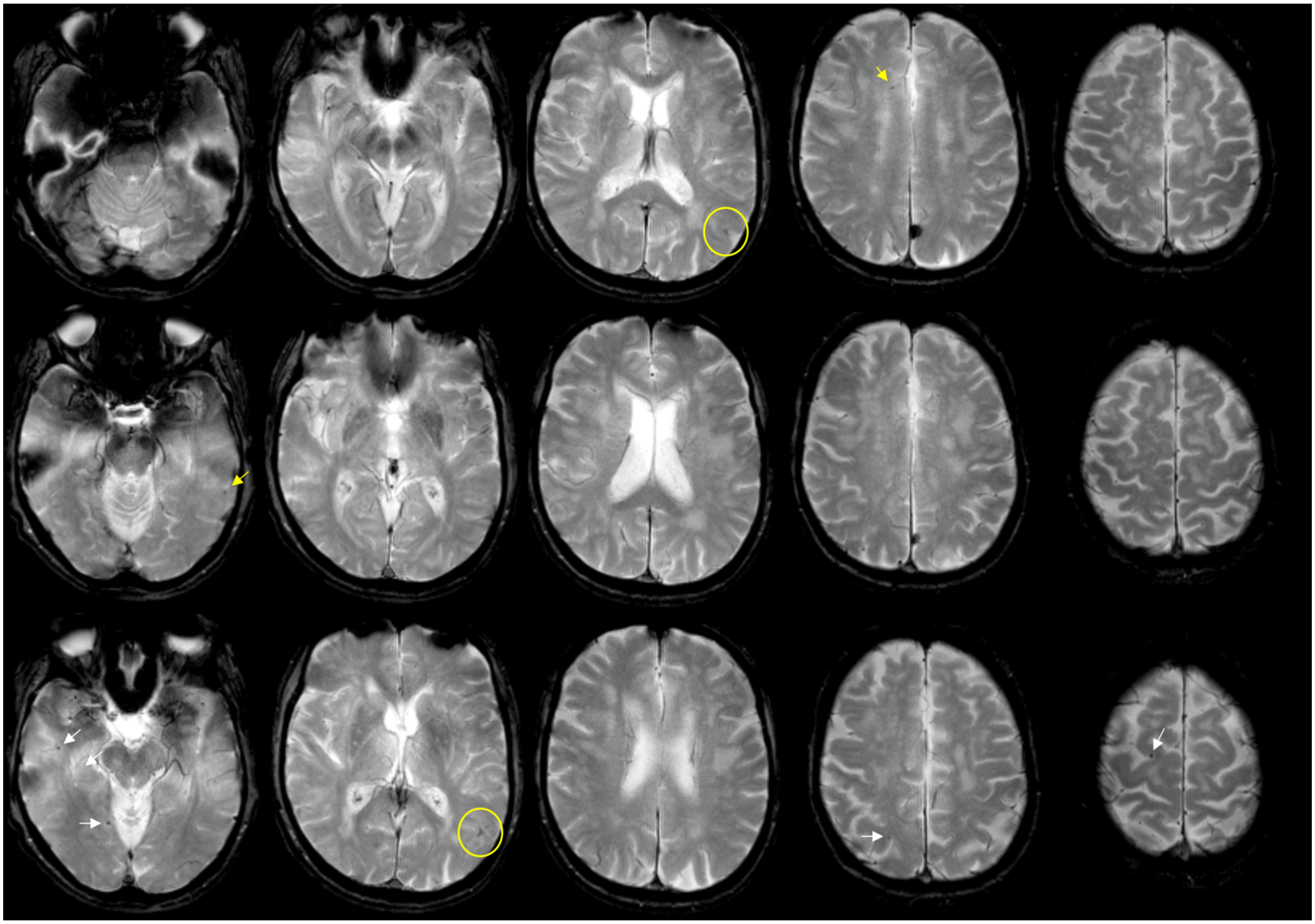

Pre-lecanemab treatment GRE-T2* imaging was obtained (3T field strength, TE 20.0, TW 639.0, slice thickness 5.0mm, interslice gap 0.5mm). Four small cerebral microhemorrhages were present in cortical or juxtacortical locations and are indicated by the white arrows. Two additional equivocal microhemorrhages are indicated by yellow arrows and an equivocal area of possible cortical superficial siderosis is present at the yellow circles. Scanning with a higher sensitivity technique like susceptibility weighted imaging and the use of a thinner slice thickness without interslice gap may have made these equivocal areas easier to interpret.

Supplementary Figure 4. Montage of GRE-T2* images from post-event MRI.

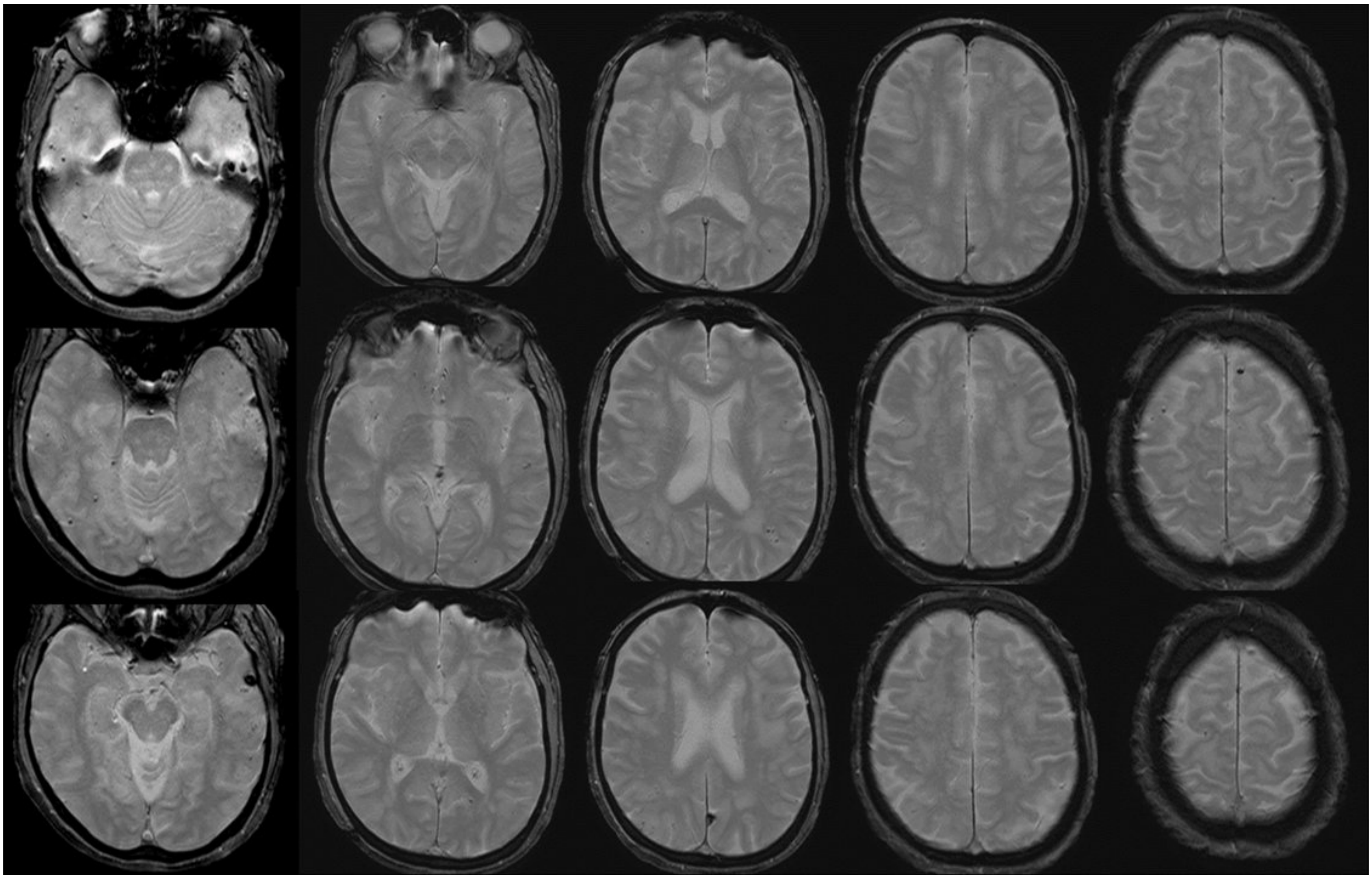

Post-lecanemab treatment GRE-T2* imaging was obtained during the patient’s hospitalization (1.5T field strength, TE 18.4, TR 671.9, slice thickness 5.0 mm, interslice gap 1.0 mm). The extent of microhemorrhagic changes greatly worsened compared to the pre-treatment imaging.

Supplementary Figure 5. Hospital acquired post-treatment selected images from diffusion weighted scan.

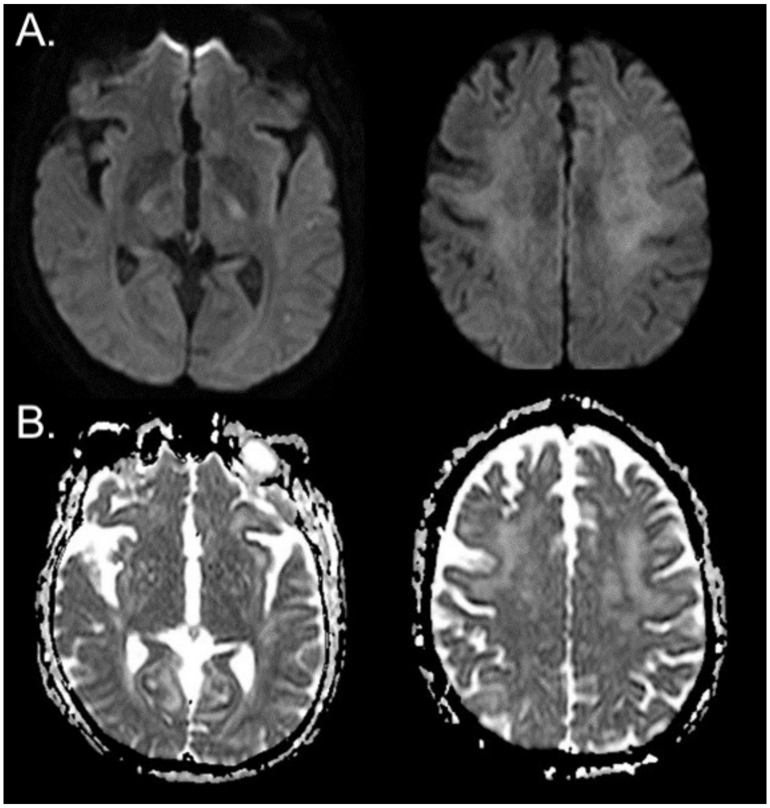

A. DWI and B. ADC images show no large areas of stroke. Two tiny foci of equivocal diffusion restriction are present in the left parietotemporal regions. There is also subtle cortical ribboning in insular cortices and frontal lobes.

Supplementary Figure 6. Selected images from florbetaben PET and flortaucipir PET imaging.

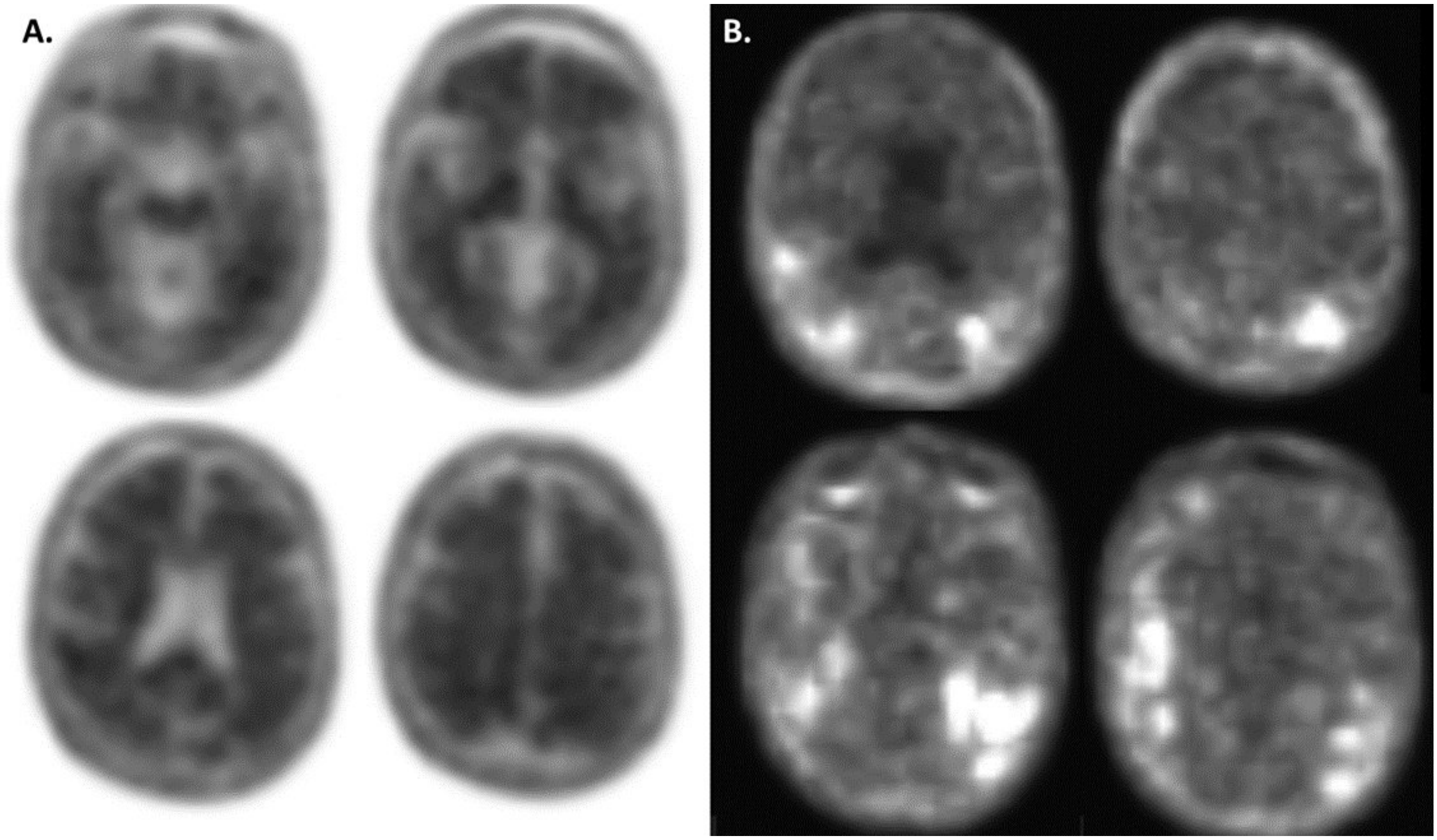

A. Montage of selected images from florbetaben PET scan obtained at enrollment in the open label extension study shows evidence of widespread cortical tracer retention consistent with considerable β-amyloid presence in the cortex.
B. Montage of selected images from the flortaucipir PET scan obtained at enrollment in the open label extension study showing tracer uptake predominantly in the temporal and parietal lobes, indicating the presence of tau pathology.

Supplementary Figure 7. Locations of tissue blocks for neuropathology examination.

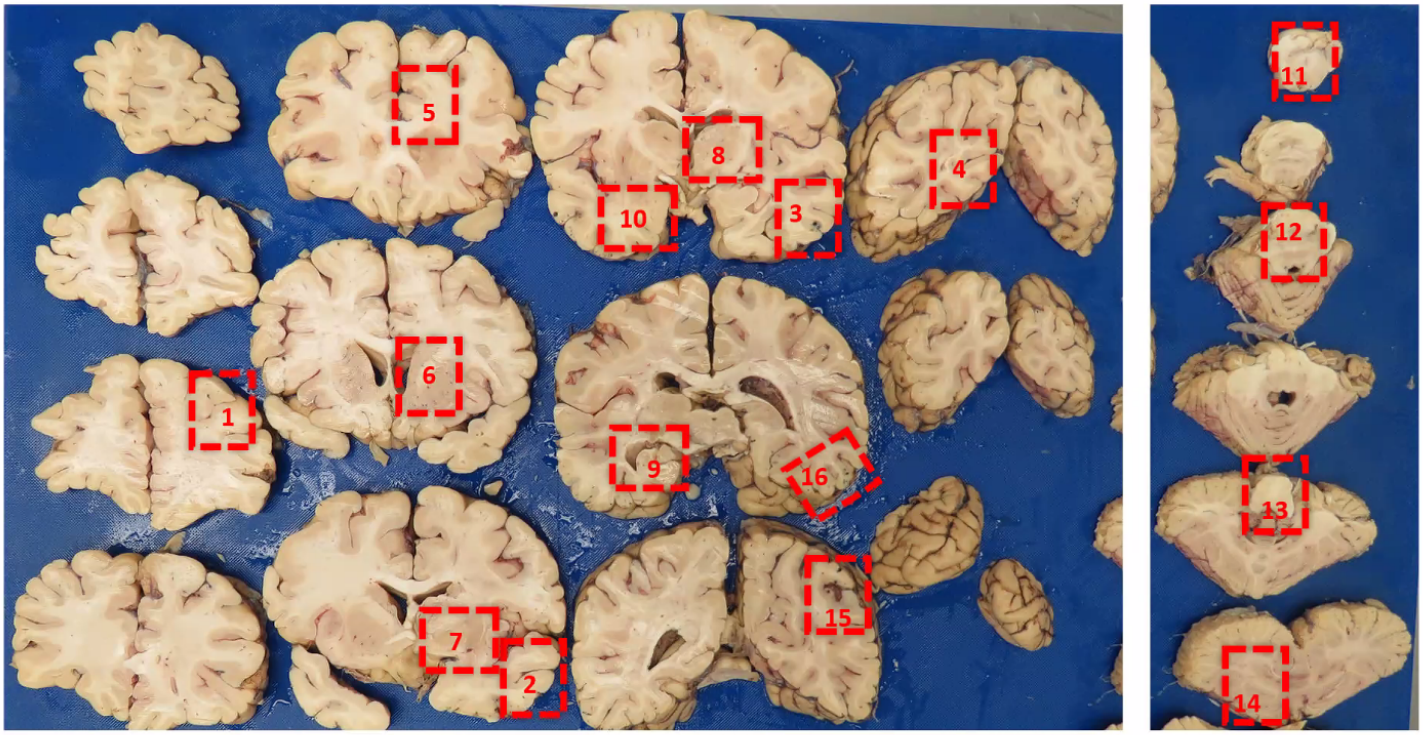

Overview image of the coronal sectioning of the brain. Red rectangles mark the tissue selected for the autopsy.

Supplementary Figure 8. β-amyloid and tau pathology.

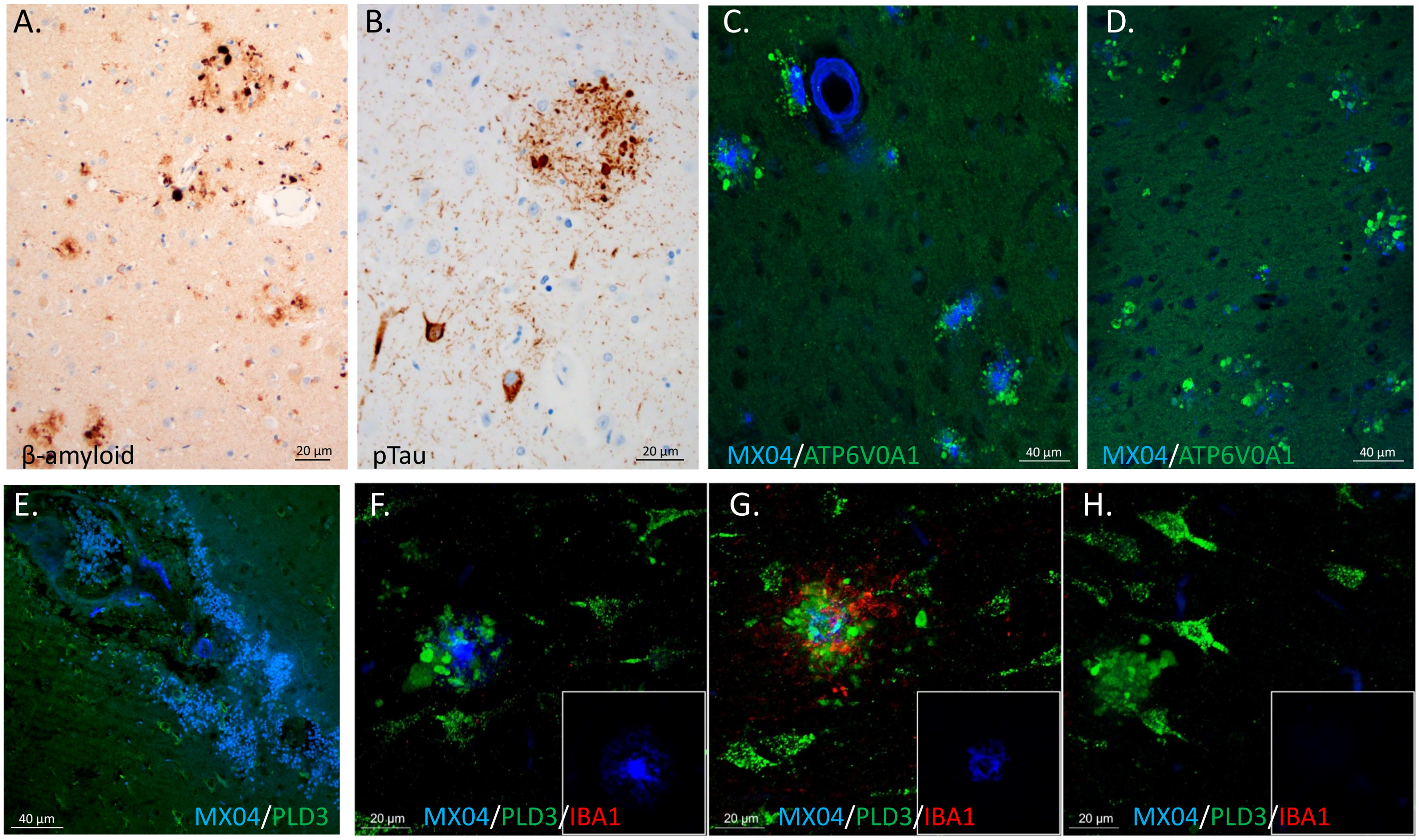

The autopsy revealed A. moderate density of β-amyloid plaques and B. a range of tau pathologies, consistent with moderate Alzheimer's disease neuropathologic changes, 20 µm scale bars. C. This frontal lobe section shows numerous plaques and vascular β-amyloid deposition, stained blue with Methoxy-X04 (MX04) and dystrophic neurites around plaques, stained green with the marker A1-subunit of the V0 domain of V-ATPases (ATP6V0A1), 40 µm scale bar. D. This section from the medial temporal lobe shows numerous rosettes of dystrophic neurites (green), some of which lack central β-amyloid staining entirely and others with faint staining, 40 µm scale bar. E. A ruptured microaneurysm on a vessel with heavy β-amyloid deposition, 40 µm scale bar. F. A normal appearing plaque with a dense core stained brightly in blue is surrounded by a halo of dystrophic stained with the neuronal lysosome marker phospholipase D3 (PLD3), 20 µm scale bar. G. A plaque that appears partially cleared (residual dense core remains present) is also surrounded by dystrophic neurites along with extensive microgliosis; microglia stained with Ionized calcium Binding Adaptor molecule 1 (IBA1) in red, 20 µm scale bar. H. A halo of dystrophic neurites without detectable β-amyloid is likely a site of β-amyloid clearance. Images F-H are z-stacks covering 15 µM in the z-plane with images every 3 µM to ensure the core of the halo of dystrophic neurites is well-captured, 20 µm scale bar (G is the same plaque as in figure 2d). Micrographs were obtained of 110 clusters of dystrophic neurites in the temporal lobe, hippocampus and parietal lobe, of which, 21% lacked central amyloid staining and 24% had faint residual staining.
