# Supplementary material for: Fatal iatrogenic cerebral β-amyloid-related arteritis in a woman treated with lecanemab for Alzheimer’s disease
Source: Nat Commun. 2023 Dec 12;14:8220. doi: 10.1038/s41467-023-43933-5 (PMC10716177; doi:10.1038/s41467-023-43933-5)
Supplement: Supplementary file 3 — Description of Additional Supplementary Files [file 41467_2023_43933_MOESM3_ESM.pdf]

Description of Additional Supplementary Files

File Name: Supplementary Movie 1
Description: This video shows three-dimensional reformatted images obtained from light sheet microscopy of a cleared block of tissue from the temporal lobe with dimensions of approximately 2 x 1.5 x 1cm. The surface of the block is rendered in grey in the beginning of the video with meningeal arteries visible. Epifluorescence images in purple show the morphology of the parenchymal vessels. Numerous microaneurysms are visible. Cloudy background staining around several vessels is due to autofluorescence from blood; rupture arterioles are visible inside several of these areas of bleeding (the video zooms in on an example at around the 40 second mark). The microvascular reconstruction was done using Imaris software. 1,000 µm bar added in the bottom left corner.

File Name: Supplementary Movie 2
Description: These three-dimensional images of arterioles associated with hemorrhages were obtained with light sheet microscopy of cleared human brain specimens. The first vessel shown is stained with thiazine red (in red, staining β-amyloid) and isolectin (in green, staining the vessel). The area of hemorrhage appears somewhat yellow due to autofluorescence. The second vessel is stained similarly. The pink channel shown at the end of the video shows only autofluorescence (no primary antibody), highlighting the location of blood. In both ruptured vessels deposits of amyloid are present at the site of rupture. 50 µm bar added in the bottom left corner.
